# A Model of Social Media Effects in Public Health Communication Campaigns: Systematic Review

**DOI:** 10.2196/46345

**Published:** 2023-07-14

**Authors:** James Kite, Lilian Chan, Kathryn MacKay, Lucy Corbett, Gillian Reyes-Marcelino, Binh Nguyen, William Bellew, Becky Freeman

**Affiliations:** 1 Prevention Research Collaboration Sydney School of Public Health The University of Sydney Camperdown Australia; 2 Sydney Health Ethics Sydney School of Public Health The University of Sydney Camperdown Australia; 3 Sydney School of Public Health The University of Sydney Camperdown Australia; 4 The Daffodil Centre The University of Sydney and Cancer Council New South Wales Camperdown Australia

**Keywords:** awareness, behavior change, campaign development, campaign evaluation, engagement, hierarchy of effects, social media, systematic review

## Abstract

**Background:**

Social media platforms are frequently used in health communication campaigns. Common understandings of campaign effects posit a sequential and linear series of steps from exposure to behavior change, commonly known as the hierarchy of effects model (HOE). These concepts need to be reevaluated in the age of social media, which are interactional and communal.

**Objective:**

This review aims to update the traditional HOE for health communication campaigns in the context of social media, including identifying indicators of effectiveness and how these are conceptualized to lead to health-related outcomes.

**Methods:**

We conducted a systematic review of studies following PRISMA (Preferred Reporting Items for Systematic Reviews and Meta-Analyses) guidelines reporting on the use of social media as part of health communication campaigns, extracting campaign information such as objectives, platforms used, and measures of campaign performance. We used these data, combined with our understanding of the HOE, to develop an updated conceptual model of social media campaign effects.

**Results:**

We identified 99 eligible studies reporting on 93 campaigns, published between 2012 and 2022. The campaigns were conducted in over 20 countries, but nearly half (n=42) were conducted in the United States. Campaigns targeted a variety of health issues and predominantly used Facebook, Twitter, Instagram, and YouTube. Most campaigns (n=81) set objectives targeting awareness or individual behavior change. Process measures (n=68; eg, reach and impressions) and engagement measures (n=73; eg, likes and retweets) were reported most frequently, while two-fifths (n=42) did not report any outcomes beyond engagement, such as changes in knowledge, behavior, or social norms. Most campaigns (n=55) collected measures that did not allow them to determine if the campaign objective had been met; that is, they were process evaluations only. Based on our review, our updated model suggests that campaign exposure can lead to individual behavior change and improved health outcomes, either through a direct or indirect pathway. Indirect pathways include exposure through social and policy changes. “Engagement” is positioned as critical to success, replacing awareness in the traditional HOE, and all types of engagement are treated as equal and good. No consideration is being given to potential negative engagement, such as the distribution of misinformation. Additionally, the process is no longer linear and sequential, with circular pathways evident, such as engagement not only influencing behavior change but also generating additional exposure to campaign messages.

**Conclusions:**

Our review has highlighted a change in conventional understandings of how campaigns can influence health outcomes in the age of social media. The updated model we propose provides social media campaigners with a starting point to develop and tailor campaign messages and allows evaluators to identify critical assumptions to test, including the role and value of “engagement.”

**Trial Registration:**

PROSPERO CRD42021287257; https://www.crd.york.ac.uk/prospero/display_record.php?RecordID=287257

## Introduction

**Figure 1:**
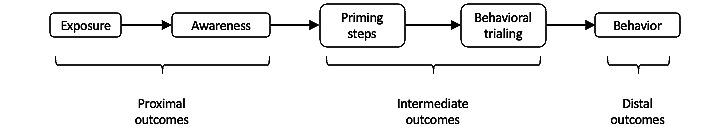
Typical hierarchy of effects model in a health campaign.

Health communication campaigns usually operate on the basic assumption that the audience response will follow a sequential and linear series of steps from exposure to the campaign to an action or behavior [1]. This process is commonly known as the hierarchy of effects model (HOE). According to HOE, exposure to a campaign message will lead to an action or change of behavior through some intermediate steps, such as change in attitudes or beliefs (Figure 1). Further, the probability of achieving each outcome is theorized to decrease as the process moves through the hierarchy, meaning that, ultimately, only a small proportion of the audience exposed to a campaign will engage in the desired behavior. However, empirical evidence demonstrating the underlying assumptions of HOE is limited, especially in relation to public health campaigns [2]. Additionally, HOE is not without its critics. Hornik and Yanovitzky [3], for example, have argued that its basic assumption, that exposure leads directly to behavior change, does not capture the full effects of a campaign. They argue that indirect influences on behavior change, such as through news media pressure or policy change, must be considered. Outside of public health, commercial marketers have also debated the merits of HOE, with some contending that it has been used largely because individual constructs, such as awareness of a campaign, are easily measured rather than because it is a valid model [4]. Others have countered that this simply means more testing is needed, and not that the model is fundamentally flawed [5]. Despite these criticisms, there is agreement that understanding how communication campaigns influence behavior is essential for effective campaign design and rigorous evaluation. Having a model like HOE allows campaign planners to consider the steps within the hierarchy and target and tailor their messages accordingly. It also allows evaluators to identify the necessary measures by which to judge a campaign’s effectiveness. In short, having a clearly defined expectation for how a campaign will work is useful for both planning and evaluation.

Much of this debate around HOE relates to campaigns using “old” media, such as television. However, over the past 2 decades, social media have become increasingly important and commonly used for health communication [6]. “Old” media channels may be characterized as “transactional” in that they assume that the communication process between campaigner and audience is linear, sequential, and based on 1-way communication of messages. Thus, these channels align with the HOE model. Social media, however, is inherently communal, allowing for multidirectional and wider sharing of messages, meaning that social media may be characterized as “interactional.” Instead of campaign awareness, practitioners are now seeking “engagement” from their audiences. Broadly speaking, engagement includes any features or functions of social media that allow users to interact, share, and create content with their social networks [7]. These features include direct dialogue between audiences and campaigners, as well as audiences sharing the campaign messages with their social networks, either directly through powerful word-of-mouth marketing or indirectly through the algorithms social media platforms use to determine what content to feed a given user. This dialogue and amplification may be generated in a more effective manner than in “old” media channels, or at least in a more measurable manner [8]. Further, there are risks that come with social media use that may result in undesirable or even harmful effects, such as the spreading of misinformation and facilitation of stigma and abuse [9,10]. The traditional HOE model does not account for these interactions and effects.

The development of social media campaign practice has outpaced that of theoretical and conceptual development. Communications theories do exist and can be useful in understanding how messages are disseminated and why people engage with social media. The One-step, Two-step, and Multi-Step Flow theories, for example, can be useful in conceptualizing the relationship between social media users and mass media [11], while the Uses and Gratification Theory helps in understanding why social media users seek out the information that they do [12]. Behavior change theories, like the Health Belief Model [13] and Diffusion of Innovations [14], are also useful in identifying the factors that influence behavior change that campaign messages can target. However, communications and behavior change theories are not sufficient when it comes to understanding the effects of social media campaigns and the assumptions that underpin campaigns. The HOE model may be able to fill this gap but given the vastly different nature of social media compared to traditional media channels, it must be reconsidered and perhaps updated or even replaced entirely. Key questions relating to campaign practice remain unanswered, especially in relation to the impact on health-related outcomes. This includes an assessment of the value of engagement in achieving campaign goals. In commercial marketing, there is limited evidence that engagement is associated with increased purchase intentions, income, and sales [15], but within health communication, it remains unclear as to what role engagement plays, if any, or whether different types of engagement are better than others and in what contexts. As mentioned above, audience engagement with a social media campaign has been framed as desirable [16], but it remains unclear what its value is. This makes it difficult for campaigners and evaluators to develop and evaluate campaigns. Amidst calls for new methods and research into digital communications [17], we need to consider the theory of how social media “works” in health communication campaigns, including the position and role of engagement. Failure to do so risks wasting resources and, worse, campaigns having a negative impact on health outcomes.

In this systematic review, we aimed to update the traditional HOE for health communication campaigns in the context of social media. Specifically, we asked:

1. What indicators are used to evaluate the effectiveness of health-related social media campaigns?
2. How are these indicators conceptualized to lead to health-related outcomes?

We were not seeking to quantify the magnitude of effects of social media on health-related outcomes or test the pathway. Instead, we hope to inform further practice-relevant research and evaluations of the use of social media in health communication.

## Methods

### Overview

We undertook a systematic review of studies following PRISMA (Preferred Reporting Items for Systematic Reviews and Meta-Analyses) guidelines (see Multimedia Appendix 1) reporting on the use of social media for health communication purposes (PROSPERO Registration: CRD42021287257). We searched 5 electronic databases (CINAHL, Scopus, MEDLINE, PsycInfo, and Web of Science) from 2007 until November 22, 2022. These databases were selected because they are the predominant databases for health-related research. The search strategies are shown in Multimedia Appendix 2.

### Eligibility and Screening

To be eligible, studies needed to be descriptions or evaluations of public health marketing campaigns that used any social media, including as part of a wider mass media or social marketing campaign, and published from 2007 onward. This year was selected because it was the first complete year in which Facebook was available to the global public. Social media metrics had to be reported separately from any other channels. “Social media” included any digital platform that enables or facilitates the creation of web-based communities for the purpose of sharing information, opinions, and content (eg, Facebook, Twitter, Instagram, WeChat, and YouTube), including purpose-built platforms. A “campaign” was defined as any sustained, deliberate effort to communicate a message or group of related messages that aim to inform, motivate, or persuade a nonclinical population. This included one-off or repeat campaigns that are continuous or episodic. We used English search terms but did not restrict eligibility by language. There were no restrictions on health issues, study design, or evaluation indicators.

Studies were excluded if they were commentaries, dissertations, or conference abstracts. Reviews were also excluded because they report on multiple campaigns collectively, potentially obscuring different conceptual pathways of effects. We also excluded studies of exposure to commercial marketing on social media, clinical interventions or health programs that used social media as a setting or delivery mechanism, campaigns that targeted nonhealth issues, studies of user experience on social media, and papers that reported only formative research for social media campaigns.

After removing duplicates, 1 author screened abstract and titles for eligibility. Two authors then independently screened the full text for the retained studies, with discrepancies resolved by discussion. The agreement between reviewers was 74% (Cohen coefficient=0.42).

### Data Extraction

We developed and pilot-tested data extraction forms, extracting all information described in Table 1. This included key campaign information, such as goals and objectives, social media platforms used, and theories and frameworks used. Goals and objectives were classified as targeting either awareness-raising, individual behavior change, or social change. Awareness-raising campaigns sought to increase awareness of a health issue (eg, mental health stigma) without explicitly aiming to change behavior. Individual behavior change campaigns aimed to change health-related behaviors of individuals in a population (eg, reducing alcohol consumption), while social change campaigns aimed to build support for wider social change (eg, adoption of supportive breastfeeding policies in workplaces). Similarly, we classified theories as individual-level, interpersonal-level, and community-level. Individual-level theories conceptualized behavior within an individual (eg, health belief model and the transtheoretical model of behavior change). Interpersonal-level theories conceptualized how social factors, such as social norms and interactions, interacted with individual-level factors and influenced the behavior of individuals (eg, dynamic transactional model and social cognitive theory). Community-level theories conceptualized how information, norms, and behaviors were transferred across and through groups (eg, diffusion of innovations and Two-step flow model of communication). Bespoke models, such as campaign-specific logic models, and frameworks for practice, such as social marketing, were also noted, but we did not classify these. Campaigns could have objectives or use theories that fit into more than one of our categories.

**Table 1:** Information extracted from included studies.

Field name	Description
Campaign name	Name or slogan [tagline] of the campaign
Citations	All relevant citations for this campaign
Country/region	Geographic bounds of the campaign
Target population	Primary and secondary target populations for the campaign
Campaign goals/objectives	What the campaign intended to achieve, including any behavior change or nonbehavior change outcomes.
Campaign delivery	Dates and duration of the campaign, including details on any phases of campaigns
Social media platforms used	The social media platforms used by the campaign
Audience interaction	Did the campaign actively encourage audience interaction? If so, in what form?
Type of campaign	Whether the campaign was: (1) “social media only” (ie, all campaign activity took place on one or more social media platforms), (2) digital only (ie, all campaign activity took place on digital channels, including but not limited to social media), (3) part of a broader stand-alone mass media campaign (ie, including nondigital media like TV), or (4) part of a comprehensive social marketing campaign (ie, including initiatives designed to address other elements of the social marketing mix, like policy change, environmental changes, or community-based initiatives)
Models and frameworks	Theoretical or planning framework used in the design of the campaign and its evaluation.
Step 1: process evaluation measures	Description of measures used in any process evaluation activity (eg, implementation or exposure metrics like reach and impressions)
Step 2: awareness measures	Description of measures used to assess whether target audience had seen the campaign (eg, recall and exposure source) and target audience response (eg, perceived relevance and believability)
Step 3: engagement measures	The type of measures for “engagement,” eg, likes, shares, and comments
Step 4: priming steps measures	Measures of knowledge, attitudes, beliefs, or intentions
Step 5: behavioral trialing measures	Measures of trialing behaviors and antecedents of behaviors (eg, calling help line and quit smoking attempt)
Step 6: outcome evaluation	Measures of the targeted outcome (eg, behavior change and policy change)

We extracted the reported measures or indicators of campaign performance or effectiveness, grouping them according to a conceptual framework based on the HOE that was developed by Chan et al [18]. This framework included 6 steps, shown in Table 1, that were used to group measures or indicators collected in the evaluations. Data were tabulated by campaign to facilitate comparison between campaigns, as opposed to studies. Two authors completed the extraction process. Data from a subset (10%) of the included studies were extracted by both authors to test for shared understanding of the data fields, with discrepancies resolved through discussion and the extraction forms amended as appropriate. Data from the remaining studies were extracted independently.

### Analysis and Development of Conceptual Pathway of Effects

We analyzed the extracted data narratively. Specifically, we considered the nature of the campaign goals, objectives, theories, and frameworks (where used), and the evaluation indicators collected and reported and the relationship between these. This included examining who and what the campaigns targeted (eg, individual behavior change and social change) and whether the reported measures aligned with the stated goal. Similarly, we compared the reported measures with the concepts of the theories and frameworks that underpinned the campaigns (whether a campaign that used the health belief model collected measures of perceived susceptibility, self-efficacy, etc).

This analysis was used to develop an initial conceptual model of social media effects by mapping the constructs that underpinned the campaigns, whether these were made explicit or implied. This initial model was reviewed as a team and built iteratively through discussion, with reference back to the extracted data as needed.

## Results

### Characteristics of Included Campaigns

**Figure 2:**
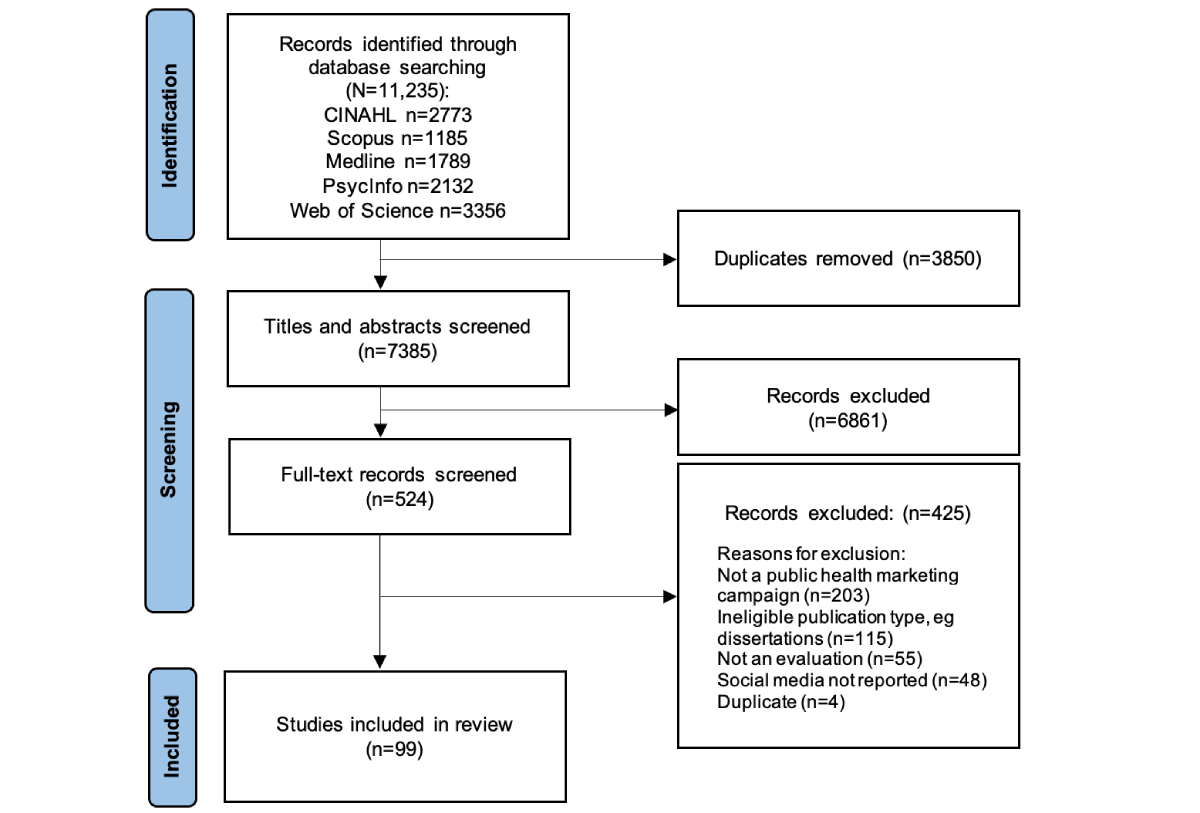
Flowchart of search and screening results.

From the 11,235 studies initially identified, we included 99 studies. These studies were published between 2012 and 2022 and related to 93 campaigns (Figure 2; Multimedia Appendix 3). Of these, 47 were social media only [19-66], 9 were digital only [67-75], 24 were mass media campaigns [76-104], and 13 were social marketing campaigns [105-117]. Most campaigns were conducted in the United States (n=42), 8 from each of Australia and Canada, 7 were from the United Kingdom, 4 from China, 3 from Italy, 2 from each of Aotearoa New Zealand and Qatar, and 1 from each of Belgium, Brazil, Chile, Denmark, Ghana, India, Indonesia, Malaysia, the Netherlands, Puerto Rico, Saudi Arabia, Vietnam, and Wales. Four were multicountry campaigns. Health issues targeted by the campaigns included smoking or vaping (n=14), sexual health or HIV (n=9), mental health (n=9), cervical cancer screening or HPV vaccination (n=7), COVID-19 (n=7), nutrition and eating disorders (n=7), overweight and obesity (n=5), Alzheimer disease or dementia (n=3), influenza vaccination (n=3), reproductive or antenatal health (n=2), physical activity (n=2), road safety (n=2), skin cancer prevention (n=3), breast cancer screening (n=2), and hepatitis (n=2), while 6 targeted multiple risk factors for chronic diseases. Other issues targeted by single campaigns were alcohol, bowel cancer screening, antibiotic use, type 2 diabetes, injury prevention, family planning, water safety, autism, and the Zika virus. The campaign duration varied from 1 week to 6 years.

The most popular platform used in the campaigns was Facebook (n=70), followed by Twitter (n=40), Instagram (n=27), YouTube (n=23), Snapchat (n=3), and WeChat, Sina Weibo, LinkedIn, and Tumblr (n=2 each). Other platforms used by 1 campaign included TikTok, Pinterest, MySpace, Grindr, Squirt, LINE, Zalo, and Flickr, as well as the now-defunct Vine. One campaign created its own social media platform, and 4 did not specify what platforms they used. Approximately half of the campaigns used just 1 platform (n=44) and half used more than one (n=45). Audience interaction was explicitly sought in just 24 campaigns, with the most common technique being to seek user-generated content, such as videos and photos (n=9). Other techniques used included contests, pledges, hashtags, prompting users to like and share content, and crowdsourcing the distribution of campaign material.

Almost all (n=81) campaigns set objectives related to awareness raising and individual behavior change. Just 5 campaigns set objectives aimed at social change, while the objectives of 9 campaigns were unclear.

With regard to theories and frameworks, 41 campaigns made explicit use of at least one theory or framework in their design or evaluation. The specific theories used varied significantly, but we classified 16 as being individual level, 8 as interpersonal level, and 9 as community level. A total of 14 campaigns used bespoke models or frameworks for practice. No campaign explicitly used the traditional HOE.

### Measures Collected and Comparisons to Objectives and Underlying Theories and Frameworks

We found that 68 campaigns had reported process measures (relating to reach, impressions, counts of posts, etc), 24 awareness measures, 73 engagement measures, 30 priming steps measures, 16 behavioral trialing measures, and 25 outcome evaluation measures. No campaigns had measures that spanned all 6 steps in the Chan et al [18] model, while 5 measured 5 of the steps, 13 measured 4 steps, 19 measured 3 steps, and 45 measured 2 steps. Eleven campaigns measured only 1 step. Just under half of the campaigns (n=42) did not measure anything beyond the engagement step, while only 8 campaigns reported measures from priming steps, behavioral trialing, and outcome evaluation only and did not measure process, awareness, or engagement. Social media–only campaigns measured fewer steps on average compared to the other campaign types (2.2 compared to 3.3 for digital-only campaigns, 2.6 for mass media campaigns, and 2.9 for social marketing campaigns).

The most common process measures were views or related measures (n=29), reach (n=29), impressions (n=23), and count of posts or tweets (n=10). The most common engagement measures were likes or reactions (n=43), shares or retweets (n=40), comments (n=36), clicks or click-through rate (n=27), and number of followers or fans (n=23).

Most campaigns (n=55) did not collect measures that allowed them to determine whether the campaign objective had been met; that is, they were process evaluations only. Similarly, of the 27 campaigns that used an explicit theory or framework, only 10 collected measures that aligned with the theory or framework. For example, 6 campaigns used the Health Belief Model; while the Health Belief Model posits that there are necessary precursor steps toward behavior change such as self-efficacy and perceived susceptibility, only 2 of these campaigns reported measuring these concepts in their evaluation.

### A Model of Social Media Campaigns

**Figure 3:**
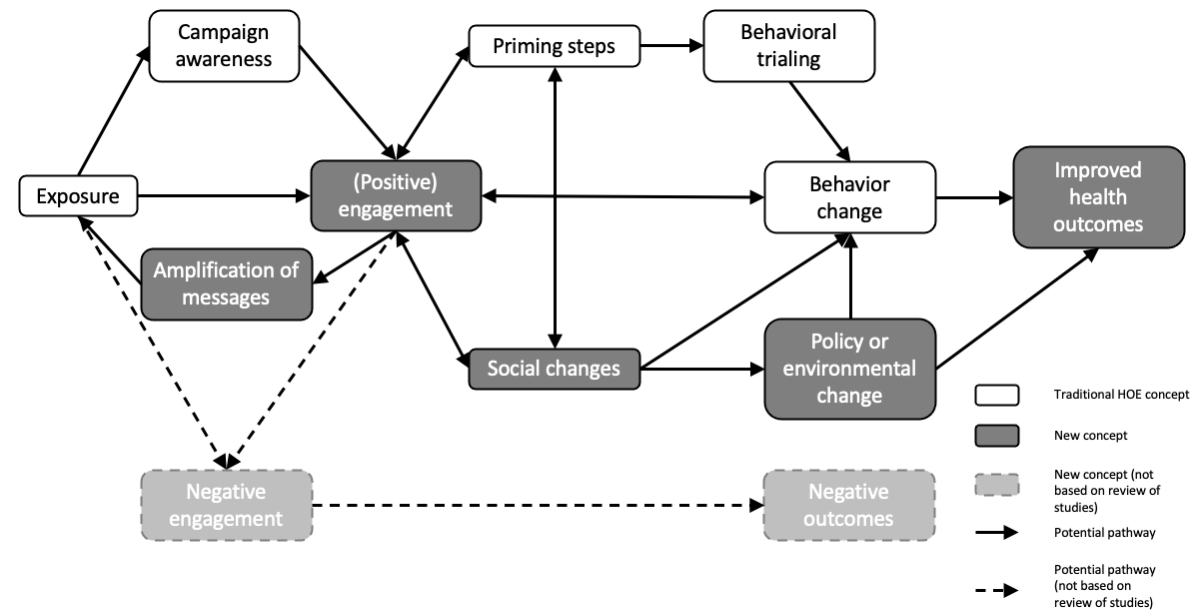
Proposed model of social media campaign effects. HOE: hierarchy of effects.

Using our review findings, we developed a model of effects for campaigns using social media (Figure 3). The model is based on a few key observations, combined with our understanding of the traditional HOE model and social media.

First, given that most campaigns set individual behavior change objectives and individual-level theories and frameworks were the most common, there appears to still be a belief that exposure to the campaign will lead directly to individual behavior change. Consistent with the conventional HOE, it was also common to position priming steps, such as attitude, knowledge, or belief change, as an important intermediate step between engagement and behavior change. However, this was not always apparent, suggesting that there is an assumption that there is a direct path from engagement to behavior change or that engagement itself is representative of priming steps or behavioral trialing.

Second, most social media campaign evaluations are focused on process and engagement measures. This focus suggests a significant variation from the traditional HOE. Engagement is now positioned as critical to the success of social media campaigns, where previously it was awareness. Awareness was measured infrequently, suggesting that either evaluators do not consider it relevant, that it is considered too resource-intensive to measure, or alternatively, there is an implicit assumption that engagement encompasses or is equivalent to awareness. In addition, engagement is defined in many ways, with no consistent attempt to distinguish between engagement types (eg, likes vs shares vs audience interaction). That is, all types of engagement are treated as equal in campaign evaluations.

Third, our model suggests that campaign effects no longer operate in a completely linear and sequential manner. It appears that there are multiple points at which campaign effects would circle back and influence earlier steps in the model. For example, engagement was positioned not just as a step toward behavior change but was also seen as important because of its ability to generate word-of-mouth marketing and message amplification, which in turn could increase exposure and lead to more engagement. Similarly, changes in priming steps may increase interest in a health issue, leading to an increase in engagement.

Fourth, although very few campaigns in our review targeted social change (eg, increased support for policy change and grassroots advocacy), there is also an assumption that engagement can lead to such changes. These social changes can then lead to behavior change or directly improve health outcomes. This is similar to the alternate HOE model proposed by Hornik and Yanovitzky [3], which also included alternative pathways to achieving behavior change.

Finally, campaign evaluations focused on the positive effects of social media use. However, the risks that come from negative engagement, like facilitating the spread of misinformation, also need to be considered in the model.

## Discussion

Our review shows that the traditional HOE model has certain deficiencies when it comes to describing a conceptual pathway of effects for public health social media campaigns. We propose that the model needs to reflect changes in the apparent value or contribution of existing concepts as well as add new concepts and pathways not previously described in the HOE. We hope that our model will be of use to campaign designers and evaluators. As with the traditional HOE, it allows for targeting and tailoring of campaign messages and highlights measures and indicators that can or should be captured in an evaluation. Equally, it highlights underlying assumptions in social media campaigns and raises questions as to the accuracy of those assumptions. This will allow researchers to test those assumptions and modify and improve on our initial model. In turn, this should lead to improved campaign practice in public health and beyond.

An important assumption underpinning our model and current social media campaign practice is that the nebulous concept of “engagement” is critical to success. Our model reflects the current assumption that engagement with the campaign is the focal point, with all outcomes dependent on generating engagement. Others have also noted how frequently engagement is reported in evaluations [118], and determining what content generates engagement is a frequent subject of research [119,120]. When considered alongside our findings, this suggests that engagement is sometimes seen as the ultimate goal of campaigns rather than an intermediate step to achieving health outcomes. However, with so few studies measuring concepts beyond engagement, it is difficult to assess the real importance of engagement. The focus of social media evaluations needs to shift from engagement to the other effects in the model, including priming steps, social effects, and behavior change. There is already discussion in the literature that can provide some insight into how this might be done [17,121,122].

Some have proposed that there are different levels of engagement, which vary in intensity and feeling toward social media content [123]. These different levels of engagement may lead to differential outcomes for a social media campaign, but we noted no consistent attempt at exploring this question. This may be because of an assumption that all engagement is “good.” However, it is well established that misinformation, disinformation, trolling, and other forms of interaction on social media can cause harm [9]. Our review shows that public health campaigns do not routinely consider negative engagement with the campaign and what impact it has on the audience or on the message of the campaign. Where negative engagement occurs, it is plausible that the campaign may fail to achieve its objectives or have the paradoxical effect of making some audience members worse off [124]. Public health campaigns are also known to sometimes have a “success to the successful” effect, whereby the position of better-off groups is improved by exposure to the campaign while the situation of marginalized or disadvantaged groups is left unchanged or worsened [125,126]. For example, health promotion campaigns in tobacco control and nutrition have sometimes been found to increase health inequities even while decreasing overall rates of ill-health [127]. Further research is needed to consider how health promotion campaigns on social media may have similar negative effects and how exposure to campaigns translates to real-world change. We need to explore whether all web-based engagement is equal and good. If it is not, there may be a hierarchy of types of engagement, which may be context-dependent. Research into these questions may help improve the conceptual model we have developed and ultimately improve campaign practice.

The role that social media companies play in shaping what is perceived to be important for evaluations must be considered. They provide a near-endless stream of data to campaigners, much of it related to engagement, making it easily available and analyzable, which in turn encourages evaluators to center engagement as an important measure of success without formally assessing whether this is the case. We acknowledge that just because engagement (or any other measure) is commonly reported does not mean that campaigners feel it is the most important or relevant measure. However, frequent reporting, coupled with the availability and ease of collecting engagement data, may have created a cycle that continuously inflates the apparent importance of engagement. Equally, the absence of reporting does not suggest that a step is not assumed to be present. For example, priming steps were infrequently measured, but the prominence of individual-level theories suggests that these steps are still considered important, even though they may not always be measured. Similarly, while awareness was infrequently reported by comparison to exposure and engagement, this may be because it is thought to be less meaningful on social media or that it is the same as engagement, or it may reflect the fact that it is more difficult to assess. This may change if social media companies change what data they make available to campaign planners. Meta (Facebook’s parent company), for example, already provides some businesses with an ability to measure awareness through what they call “brand lift” studies [128]. These studies select users who fit the campaign’s target audience and randomly assign them to exposure or control before surveying them on ad recall, brand awareness, and message association. Should tools like this become more widely available, we may see a shift in the number of social media health campaigns reporting awareness. All this raises the question of whether we are focusing on engagement because it matters or because it is easy to measure? In this way, we are echoing the criticisms of the traditional HOE and the primacy that that model gives to awareness [4].

We found that almost all campaigns included in our review were awareness-raising or individual behavior-change campaigns. In other words, they were not making use of the “social” element of social media. The potential for using social media to generate social movements and shift social norms and structures has been highlighted elsewhere [10], but our findings indicate that very few campaigns have attempted to realize this potential. Given this, it is possible that our model does not accurately or completely capture potential social effects and relevant pathways. More campaigns that aim to have social effects are needed, along with rigorous evaluation and reporting.

The interactional rather than transactional nature of our proposed model of social media campaign effects (Figure 3) brings to mind recent developments in the application of systems thinking to health issues [129]. The use of systems approaches is at an early stage of development, but in recent years researchers have begun to turn their attention to the application of systems thinking in social marketing [130-133] and more specifically to social media [134,135]. Our proposed model in Figure 3 is relatively simple, with tight boundaries to emphasize the newly introduced concepts. In a web-based version, our model has also been rendered as a more expanded systems map to illustrate possible future directions for this work [136]. Multimedia Appendix 4 explains the model in more detail.

A strength of this study is that we adopted a systematic approach to reviewing evidence and included campaigns from across numerous areas of public health. This should boost the generalizability of our model within health. A limitation of our study is that we did not consider scale in developing the model. Social media can be used to target small communities, as well as for mass-reach campaigns, so further research should explore whether the effects or the pathway of effects vary depending on the scale of the campaign. Additionally, while we did not exclude studies written in languages other than English, our use of English search terms would likely mean that some relevant campaigns have been missed. Similarly, as most campaigns were conducted in high-income, English-speaking campaigns, the generalizability of our model outside of these countries will need to be explored further. Finally, as research lags practice in this space, some of the most popular social media platforms (eg, Tik Tok) are underrepresented in our review. The way that campaigns work or are theorized to work on these platforms may differ from what is represented in our model. This highlights the need to regularly review and update our model as new information comes to light.

Our review shows that the traditional HOE that underpins health communication campaigns needs to be updated to reflect the nature of social media. The model we have developed is intended as a first step to addressing the shortcomings of the traditional HOE and assisting campaign planners and evaluators in developing and evaluating campaigns. Further testing of the model is essential, however, especially in relation to the role of engagement in the conceptual pathway.

## Appendix

Multimedia Appendix 1 PRISMA Checklist.

Multimedia Appendix 2 Search strategy.

Multimedia Appendix 3 Characteristics of included campaigns.

Multimedia Appendix 4 Explanatory video for systems model of social media effects.

## Abbreviations

HOE: hierarchy of effects
PRISMA: Preferred Reporting Items for Systematic Reviews and Meta-Analyses

## Data Availability

All data generated or analyzed during this study are included in this published article and the Multimedia Appendices.
