# Supplementary material for: A Model of Social Media Effects in Public Health Communication Campaigns: Systematic Review
Source: J Med Internet Res. 2023 Jul 14;25:e46345. doi: 10.2196/46345 (PMC10382952; doi:10.2196/46345)
Supplement: Multimedia Appendix 2 [file jmir_v25i1e46345_app2.docx]

## Appendix 2 – Search strategy

### Scopus

(TITLE-ABS-KEY (“social media” OR “social networking” OR “digital media” OR facebook OR twitter OR youtube OR Instagram OR wechat OR “tik tok” OR douyin OR weibo OR snapchat OR “Whats app” OR tumblr OR snapchat OR pinterest OR linkedin) AND TITLE-ABS-KEY (smoking OR obes* OR depression OR “physical*activ*” OR alcohol OR nutrition* OR diet* OR health OR overweight OR sedentar* OR cancer OR “illicit drug*”) AND TITLE-ABS-KEY (health W/6 (communicat* OR campaign* OR promot* OR adverti* OR marketing OR messag* OR educat* or disseminat*) ) AND TITLE-ABS-KEY (engag* OR effective* OR evaluat* OR assess* OR apprais* OR benefit* OR limitation* OR analys* OR analyz*) NOT TITLE-ABS-KEY (patient* OR review))

### PsycINFO

| # | Search Statement |
| --- | --- |
| 1 | exp Social Media/ |
| 2 | online social networks/ |
| 3 | ("social media" or "social networking" or "digital media" or facebook or twitter or youtube or instagram or wechat or "tik tok" or douyin or weibo or snapchat or Whats app or foursquare or yelp or Wikipedia or tumblr or snapchat or pinterest or linkdin).tw. |
| 4 | 1 or 2 or 3 |
| 5 | health promotion/ |
| 6 | health education/ or public health campaigns/ |
| 7 | marketing/ or digital marketing/ or social marketing/ |
| 8 | advertising/ |
| 9 | exp Health Information/ |
| 10 | (health adj6 (communicat* or campaign* or promot* or adverti* or marketing or messag* or educat* or disseminat* or engag*)).tw. |
| 11 | 5 or 6 or 7 or 8 or 9 or 10 |
| 12 | evaluation/ or program evaluation/ |
| 13 | feasibility stud*.tw. |
| 14 | pilot stud*.tw. |
| 15 | (engag* or effectiv* or evaluat* or assess* or apprais* or benefit* or limitation* or analys* or analyz*).tw. |
| 16 | 12 or 13 or 14 or 15 |
| 17 | exp obesity/ |
| 18 | smoking cessation/ or tobacco smoking/ |
| 19 | drinking behavior/ or alcohol drinking patterns/ or alcoholic beverages/ |
| 20 | exercise/ or physical activity/ |
| 21 | healthy aging/ |
| 22 | eating behavior/ |
| 23 | sedentary behavior/ |
| 24 | mental health/ |
| 25 | "depression (emotion)"/ |
| 26 | exp Cannabis/ or exp Drug Dependency/ or exp Alcoholism/ or exp Drug Usage Attitudes/ or exp Alcohol Abuse/ or exp Drug Usage/ or exp Drug Abuse/ or exp Drug Addiction/ |
| 27 | exp Chronic Illness/ |
| 28 | chronic illness/ or disease management/ |
| 29 | (smoking or obes* or depression or "physical* activ*" or alcohol or nutrition* or diet* or health or overweight or sedentar* or cancer or "illicit drug*").tw. |
| 30 | Healthy lifestyle*.tw. |
| 31 | 17 or 18 or 19 or 20 or 21 or 22 or 23 or 24 or 25 or 26 or 27 or 28 or 29 or 30 |
| 32 | 4 and 11 and 16 and 31 |
| 33 | patient*.tw. |
| 34 | patient*.mp. |
| 35 | 32 not 33 |
| 36 | 32 not 34 |
| 37 | Review.tw. |
| 38 | 34 not 37 |

Complete search strategies for Medline, Web of Science, and CINAHL are available on request
