# Supplementary material for: A Model of Social Media Effects in Public Health Communication Campaigns: Systematic Review
Source: J Med Internet Res. 2023 Jul 14;25:e46345. doi: 10.2196/46345 (PMC10382952; doi:10.2196/46345)
Supplement: Multimedia Appendix 3 [file jmir_v25i1e46345_app3.docx]

## Appendix 3 – Characteristics of included campaigns

| **Citation(s) and country/region** | **Campaign name** | **Health issue** | **Target population** | **Campaign type** | **Social media platform(s)** | **Use of theory/ framework** | **Step 1: Process evaluation** | **Step 2: Awareness** | **Step 3: Engagement** | **Step 4: Priming steps** | **Step 5: Behavioural trialling** | **Step 6: Outcome evaluation** |
| --- | --- | --- | --- | --- | --- | --- | --- | --- | --- | --- | --- | --- |
| Agha-Mir-Salim et al 2020; UK | SunSafe | Skin cancer prevention | 18-29 year olds | Social media only | Facebook | No |  |  |  | 🗸 |  |  |
| Ahmed et al 2018; Global | World Autism Awareness Day | Autism | Twitter users | Social media only | Twitter | No |  |  | 🗸 |  |  |  |
| Albalawi and Sixsmith 2017; Saudi Arabia | Untitled | Road safety | Not specified | Social media only | Twitter | Yes | 🗸 |  | 🗸 |  |  |  |
| Allen et al 2020; USA | Untitled | HPV vaccination | Young adult women living in public housing | Social media only | Twitter | Yes |  | 🗸 | 🗸 | 🗸 |  | 🗸 |
| An et al 2021; Qatar | Untitled | Breast cancer screening | Women aged 45 years and over | Social media only | Facebook, Instagram | Yes | 🗸 |  | 🗸 |  |  |  |
| An et al 2021; Qatar | Untitled | Influenza vaccination | People living in Qatar | Social media only | Facebook, Instagram | Yes | 🗸 |  | 🗸 |  |  |  |
| Andrade et al 2018; USA | Adelante | Chronic disease prevention | Latino immigrant youth (12-19 years) | Social marketing campaign | Facebook | Yes | 🗸 |  | 🗸 |  |  |  |
| Barchitta et al 2020; Italy | Obiettivo Antibiotico | Antibiotic use | General public and health professionals | Mass media campaign | Facebook, Instagram, LinkedIn, Twitter | No | 🗸 |  | 🗸 |  |  |  |
| Barragan et al 2014; USA | Sugar Pack | Nutrition | Adults | Mass media campaign | Facebook, Twitter, YouTube | Yes | 🗸 | 🗸 |  | 🗸 |  |  |
| Berends and Halliday 2018; Australia | Deadly Sport Gippsland | Chronic disease prevention | Not specified | Social marketing campaign | Facebook | Yes | 🗸 | 🗸 | 🗸 | 🗸 | 🗸 |  |
| Bonnevie et al 2020; USA | NJ Sugarfreed | Nutrition | New Jersey residents (18-65 years) eligible for Medicaid | Digital only | Facebook, Instagram, Twitter | Yes | 🗸 |  | 🗸 | 🗸 |  | 🗸 |
| Bonnevie et al 2021a; USA | Strong beautiful future | Low birthweight | Black women | Digital only | Facebook, Twitter, YouTube, Instagram | Yes | 🗸 |  | 🗸 | 🗸 |  |  |
| Bonnevie et al 2021b; USA | Stop Flu | Influenza vaccination | African American and Hispanic adults | Social media only | Facebook, Twitter, Instagram | Yes |  |  | 🗸 |  |  |  |
| Booth et al 2018; Canada | Bell Let’s Talk | Mental health | General public | Social media only | Twitter | No |  |  |  |  |  | 🗸 |
| Bopp et al 2018; USA | Active Lions | Physical activity | University staff and students | Social marketing campaign | Facebook, Twitter | Yes | 🗸 | 🗸 | 🗸 |  |  | 🗸 |
| Brittain et al 2018; USA | Colorectal Cancer Screening Awareness for Women | Bowel cancer screening | Women aged 45-64 years | Social media only | Facebook | No | 🗸 |  | 🗸 |  |  |  |
| Buller et al 2021; USA | Health Chat | Skin cancer prevention | Mothers with teenage daughters | Social media only | Facebook | Yes |  |  |  | 🗸 |  | 🗸 |
| Calder et al 2020; Aotearoa New Zealand | All right? Campaign | Mental health | Not specified | Mass media campaign | Facebook | Yes |  | 🗸 | 🗸 |  | 🗸 |  |
| Chan et al 2022; Australia | Shisha No Thanks | Tobacco | Young people (18-35) from an Arabic-speaking background | Mass media campaign | Facebook, YouTube, Instagram | Yes |  | 🗸 |  | 🗸 |  |  |
| Chittamuru et al 2020; USA | The Bigger Picture | Diabetes | Youth and young adults | Social media only | Facebook | No | 🗸 |  | 🗸 |  | 🗸 |  |
| Chung et al 2015, Chung 2016; USA | Tips from former smokers | Tobacco | Adult smokers | Mass media campaign | Facebook, Twitter, YouTube | Yes | 🗸 |  | 🗸 |  |  |  |
| Crankshaw et al 2022; USA | This free life | Tobacco | LGBT young adult occasional smokers | Mass media campaign | Not specified | No | 🗸 | 🗸 |  | 🗸 |  |  |
| Criss et al 2019; USA | Massachusetts Childhood Obesity Research Demonstration Study (MA-CORD) | Obesity | Low income communities | Social marketing campaign | Facebook | Yes |  |  | 🗸 |  |  |  |
| daVeiga et al 2020; Brazil | Untitled | HPV vaccination | Adolescents | Mass media campaign | Facebook | No |  |  | 🗸 | 🗸 |  |  |
| Dehlin et al 2019; USA | PrEP4Love | HIV | Black, gay, and bisexual men and other MSM and black transwomen | Mass media campaign | Facebook, Instagram | No | 🗸 |  | 🗸 |  |  |  |
| Diouf et al 2022; USA | Action minded | Mental health | Adults (18-65) | Social media only | Not specified | Yes |  | 🗸 |  | 🗸 | 🗸 |  |
| Duong et al 2021; Vietnam | Vietnam Ministry of Health’s COVID-19 prevention campaign | Covid-19 | Not specified | Social media only | Facebook, Twitter, YouTube, Instagram, Zalo | Yes |  | 🗸 |  | 🗸 |  |  |
| England et al 2021; USA | Rethink Vape | Vaping | Adolescents | Digital only | Snapchat, YouTube | No | 🗸 |  | 🗸 | 🗸 |  |  |
| Fagan et al 2020; USA | Untitled | HIV | Men who have sex with men and transgender women | Mass media campaign | Facebook | Yes | 🗸 |  |  |  | 🗸 | 🗸 |
| Ford et al 2022; Wales | #Timetobekind | Covid-19 | Not specified | Mass media campaign | Facebook, Twitter, Instagram | No | 🗸 |  | 🗸 | 🗸 |  |  |
| Friedman et al 2014; USA | GYT: Get Yourself Tested | Sexual health | Youth (25 years and under) | Social marketing campaign | Facebook, Twitter | Yes |  |  | 🗸 |  | 🗸 | 🗸 |
| Friedman et al 2016; Puerto Rico | Un Café por el Alzheimer | Alzheimer’s disease | Not specified | Social media only | Facebook | No | 🗸 |  | 🗸 | 🗸 |  |  |
| George et al 2016; USA | Untitled | Obesity | Black and Hispanic adults | Social marketing campaign | Facebook, Twitter | No | 🗸 | 🗸 |  |  | 🗸 | 🗸 |
| Glennie et al 2022; Australia | Untitled | Covid-19 | Aboriginal and Torres Strait Islander people living in regional and remote areas | Social media only | Facebook | No | 🗸 |  | 🗸 |  |  |  |
| Gold et al 2012, Nguyen 2013; Australia | The FaceSpace Project | Sexual health | 16-29 year olds and men who have sex with men | Social media only | Facebook, Flickr, Twitter, YouTube | No | 🗸 |  | 🗸 |  |  |  |
| Gough et al 2017; UK | Untitled | Skin cancer prevention | Adults | Social media only | Twitter | No | 🗸 |  | 🗸 | 🗸 |  |  |
| Grantham et al 2021; Canada | #eatwellcovid19 | Covid-19 | Saskatchewan residents | Social media only | Facebook, Twitter, Instagram | No | 🗸 |  | 🗸 |  |  |  |
| Hair et al 2017, Kostygina et al 2020, Romberg et al 2020a, Romberg et al 2020b; USA | Truth | Tobacco | Youth and young adults (15-21 years) | Mass media campaign | Twitter, YouTube | Yes | 🗸 | 🗸 | 🗸 |  | 🗸 |  |
| Halsall et al 2019; Canada | Mindyourmind | Mental health | Youth and young adults (13-25 years) | Digital only | Facebook, MySpace, Twitter, YouTube | Yes | 🗸 |  | 🗸 | 🗸 |  | 🗸 |
| Harding et al 2020; Ghana | Breastfeed4Ghana | Breastfeeding | Adults | Social media only | Facebook, Twitter | No | 🗸 | 🗸 | 🗸 | 🗸 |  |  |
| He et al 2017; China | Untitled | Obesity | Adults | Social media only | WeChat | No | 🗸 |  | 🗸 |  | 🗸 | 🗸 |
| Hou et al 2022; China | #Zika is that far and this close | Zika virus | Adults | Social media only | Sina-Weibo | No | 🗸 |  | 🗸 |  |  |  |
| Jacobs et al 2016; USA | What Should We Call Quitting | Tobacco | 18-24 year old smokers | Social media only | Tumblr | No |  |  | 🗸 |  |  |  |
| Januraga et al 2020; Indonesia | Pretty and Picky | Nutrition | Adolescent women (16-29 years) | Social marketing campaign | Facebook, YouTube, Instagram, LINE | Yes | 🗸 |  |  | 🗸 |  |  |
| Jawad et al 2015; UK | ShishAware | Tobacco | Not specified | Social media only | Facebook, Twitter, YouTube | No | 🗸 |  | 🗸 |  |  |  |
| Jiang and Beudoin 2016; China | China Tobacco Control Media Campaign | Tobacco | General public | Social media only | Sina Weibo | No | 🗸 |  | 🗸 |  |  |  |
| John et al 2017; USA | Choose 1% Milk | Nutrition | Adults | Social marketing campaign | Facebook | No | 🗸 |  | 🗸 |  |  |  |
| Kim and Kim 2020; USA | CDC and Prevention | Chronic disease prevention | Not specified | Social media only | Instagram | No | 🗸 |  | 🗸 |  |  |  |
| Kite et al 2018, Kite et al 2019; Australia | Make Healthy Normal | Obesity | Adults | Mass media campaign | Facebook | No | 🗸 | 🗸 | 🗸 |  | 🗸 |  |
| La Torre et al 2020; Italy | Ma che sei scemo? Il fumo fammale | Tobacco | Smokers | Mass media campaign | Facebook, YouTube | No | 🗸 |  | 🗸 |  |  |  |
| Lam and Woo 2020; USA | Untitled | Dementia | Chinese American adults (45+ years) | Social media only | YouTube | No | 🗸 |  | 🗸 |  |  |  |
| Latha et al 2020; India | Buddies for Suicide Prevention; #Iquittobacco; #Migrainethepainfultruth | Mental health | Not specified | Social media only | Facebook, Instagram | No | 🗸 |  | 🗸 |  |  |  |
| Lenoir et al 2017; UK | Smear for Smear | Cervical cancer screening | Women under 45 years | Social media only | Twitter | No | 🗸 |  | 🗸 |  |  |  |
| Liang et al 2020; China | HIV/AIDS prevention tips | HIV | Adults | Social media only | WeChat | Yes |  |  |  | 🗸 |  | 🗸 |
| Lister et al 2015; USA | Utah Family Meals | Nutrition | Youth and young adults (13-25 years) | Social media only | Facebook, Instagram, Pinterest, Twitter | Yes | 🗸 |  | 🗸 |  |  |  |
| Livingston et al 2012; Canada | In one voice | Mental health | Women 25-64 years | Mass media campaign | Facebook, Twitter, YouTube | No |  | 🗸 | 🗸 | 🗸 | 🗸 | 🗸 |
| Loft et al 2020, Pedersen et al 2020; Denmark | Stop HPV – stop cervical cancer | HPV vaccination | Mothers hesitant about HPV vaccination of their daughters | Social media only | Facebook | No | 🗸 | 🗸 | 🗸 |  |  |  |
| Lucini et al 2020; Italy | Stay home stay fit | Chronic disease prevention | Not specified | Digital only | Facebook, Twitter, YouTube | No | 🗸 |  | 🗸 |  |  |  |
| Lyson et al 2019; USA | Untitled | Cervical cancer screening | Not specified | Social media only | Bespoke platform | No |  |  |  | 🗸 |  | 🗸 |
| Maafs-Rodriguez et al 2022; Chile | El Plato para Comer Saludable para Ninos | Nutrition | Spanish-speaking children | Social media only | Facebook, Twitter, Instagram | Yes | 🗸 |  | 🗸 |  |  |  |
| Marcell et al 2022; USA | One Vax Two Lives | Covid-19 | Pregnant and lactating women | Social media only | Facebook, Twitter, Instagram, TikTok | Yes | 🗸 |  | 🗸 |  |  |  |
| Mat Johar et al 2021; Malaysia | Burn and Blast Awareness | Injury prevention | Adults | Social media only | Facebook, YouTube, Instagram | No | 🗸 |  | 🗸 |  |  | 🗸 |
| Miller et al 2022; USA | Untitled | Tobacco | Women (18-45) | Social media only | Facebook | No | 🗸 |  | 🗸 |  |  |  |
| Mohanty et al 2018; USA | 3forME | HPV vaccination | Adolescents | Social media only | Facebook | Yes | 🗸 | 🗸 | 🗸 |  |  | 🗸 |
| Nadarzynski et al 2019; UK | Untitled | Sexual health | 13-25 year olds | Social media only | Facebook | No | 🗸 |  | 🗸 |  | 🗸 | 🗸 |
| Namkoong et al 2018; USA | Untitled | Tobacco | College students | Social media only | Facebook | No |  |  |  | 🗸 |  |  |
| Parackal et al 2017; Aotearoa New Zealand | Don’t know? Don’t drink | Alcohol | Women 18-30 years | Social media only | Facebook | Yes | 🗸 |  | 🗸 |  |  |  |
| Plant et al 2020; USA | Test4HepC | Hepatitis C | Baby boomers | Digital only | Facebook | Yes | 🗸 | 🗸 | 🗸 |  | 🗸 | 🗸 |
| Ponce-Gonzalez et al 2021; USA | Untitled | Influenza vaccination | Latinx migrant and refugees | Social marketing campaign | Facebook, Twitter, YouTube, Instagram, LinkedIn | No | 🗸 |  | 🗸 | 🗸 |  |  |
| Post et al 2013; USA | Smokefree Women | Tobacco | Women smokers | Social media only | Facebook | No | 🗸 |  | 🗸 |  |  |  |
| Rayward et al 2019; Australia | 10,000 Steps Australia Program | Physical activity | Adults | Digital only | Facebook, Instagram | Yes | 🗸 |  | 🗸 |  | 🗸 | 🗸 |
| Reuter et al 2021; USA | Untitled | Tobacco | English-speaking social media users | Social media only | Facebook, Twitter, Instagram | Yes |  |  | 🗸 |  |  |  |
| Ross et al 2016; Canada | Untitled | Sexual health | Men who have sex with men | Digital only | Facebook, Grindr, Squirt | No | 🗸 |  |  |  |  | 🗸 |
| Sampogna et al 2017; UK | Time to change | Mental health | 25-45 year olds from middle income group | Social marketing campaign | Facebook, Twitter | No |  | 🗸 | 🗸 | 🗸 |  |  |
| Sanders et al 2018; USA | Smokefree Teen | Tobacco | Teenagers | Mass media campaign | Facebook, Twitter, YouTube | Yes | 🗸 |  | 🗸 |  | 🗸 |  |
| Santarossa et al 2018; Canada | LancerHealth | Chronic disease prevention | University staff and students | Mass media campaign | Instagram, Twitter | Yes |  |  | 🗸 |  |  |  |
| Schlichthorst et al 2018; Australia | Man up | Mental health | Men | Mass media campaign | Facebook, Instagram, Tumblr, Twitter, YouTube | No | 🗸 |  | 🗸 |  |  |  |
| Schwartz et al 2022; USA | Stick it to Hep A | Hepatitis A | Adults | Social media only | Facebook | Yes | 🗸 |  | 🗸 |  |  |  |
| Silva et al 2022; Burkina Faso, Côte d’Ivoire, Niger, and Togo | Merci Mon Heros (MMH) | Family planning | Youth and young adults | Mass media campaign | Facebook, Twitter, YouTube, Instagram | No | 🗸 |  | 🗸 |  |  |  |
| Smith et al 2021; Canada | Preventable | Water safety | Recreational boaters | Social marketing campaign | Facebook | No | 🗸 |  | 🗸 |  |  |  |
| Stewart et al 2020; Canada | Buckle up; Phone down | Road safety | 16-44 year olds | Social marketing campaign | Facebook, Twitter, YouTube | No | 🗸 | 🗸 | 🗸 | 🗸 |  | 🗸 |
| Strekalova and Damiani 2018; USA | Tobacco Free Florida | Tobacco | Smokers | Social media only | Facebook | Yes | 🗸 |  | 🗸 |  |  |  |
| Suarez-Lledo et al 2021; USA | NEDAwareness | Eating disorders | Youth and young adults | Social media only | Twitter | Yes | 🗸 |  | 🗸 |  | 🗸 |  |
| Sundstrom et al 2018; USA | It’s my time | Cervical cancer screening | College-age women | Mass media campaign | Facebook, Twitter, Vine | Yes | 🗸 | 🗸 | 🗸 | 🗸 |  | 🗸 |
| Syred et al 2014; UK | Say yes to the test | Sexual health | Young people (15-24) | Mass media campaign | Facebook | No | 🗸 |  | 🗸 |  |  |  |
| Tesoriero et al 2022; USA | PrEP Aware Week | HIV | Not specified | Mass media campaign | Facebook, Twitter, Instagram, Snapchat | No |  |  |  |  |  | 🗸 |
| Theiss et al 2015; USA | Know: BRCA; Bring your Brave | Breast cancer screening | Women under 45 | Social media only | Facebook | No | 🗸 |  | 🗸 |  |  |  |
| Tully et al 2019; Australia | The Father Effect | Mental health | Fathers | Mass Media campaign | Not specified | Yes |  | 🗸 |  | 🗸 |  |  |
| Van Asbroeck et al 2021; Belgium | SaniMemorix | Dementia | Adults (40-75) | Mass media campaign | Not specified | No |  | 🗸 |  | 🗸 |  | 🗸 |
| Vandeormael et al 2021; USA, Mexico, UK, Germany, and Spain | CoVideo | Covid-19 | Adults (18-59) | Social media only | Facebook, Twitter, YouTube, Instagram | Yes |  |  |  | 🗸 |  |  |
| Wagner et al 2022; USA | Swap Up | Overweight and obesity | Teenagers | Mass media campaign | Facebook, Instagram | Yes | 🗸 | 🗸 | 🗸 |  |  | 🗸 |
| Yousuf et al 2020; Netherlands | Covid-19 hygiene | Covid-19 | Not specified | Digital only | YouTube | Yes | 🗸 | 🗸 |  |  |  | 🗸 |
| Zaidan et al 2015; Middle East | Prevention is better than cure | Chronic disease prevention | Middle Eastern population | Social marketing campaign | Facebook | No | 🗸 |  | 🗸 |  |  |  |
